# Doxycycline versus prednisolone as an initial treatment strategy for bullous pemphigoid: a pragmatic, non-inferiority, randomised controlled trial

**DOI:** 10.1016/S0140-6736(17)30560-3

**Published:** 2017-04-22

**Authors:** Hywel C Williams, Fenella Wojnarowska, Gudula Kirtschig, James Mason, Thomas R Godec, Enno Schmidt, Joanne R Chalmers, Margaret Childs, Shernaz Walton, Karen Harman, Anna Chapman, Diane Whitham, Andrew J Nunn, UK Dermatology Clinical Trials Network BLISTER Study Group, J Adams, V Akhras, A Anstey, C Barnard, H Bell, S Blackford, E Bröcker, A Carmichael, R R Coelho, F Craig, K Davies, R Ellis, J English, R Gläser, R Groves, C Günthert, P J Hampton, N Hepburn, R Hügel, K Hussain, J Ingram, A M Layton, N J Levell, V Lewis, H Malhomme, A Omerod, G Patel, R Rallan, J Ravenscroft, H Santander, K Steinbrink, M Sticherling, C Thomas, M Vatve, N van Beek, V Venning, E Veysey, R Wachsmuth, S Wahie, B Walker, M Walsh, J Wee, M Westmoreland, G Wong, Adam Ferguson, Indre Verpetinske, Emilia Duarte-Williamson, Fiona Antony, Chris Bower, David Gawkrodger, Kathy Taghipour, M G S Dunnill, Alex Waters, Walter Bottomley, Andrew Wright, Jane Sterling, Adzura Azam, Sam Gibbs, Thomas Luger, Ingrid Salvary, Chris Lovell, Andrew Ilchyshyn, Karen Gibbon, Marinella Nik, Robert Charles-Holmes, A Lloyd Lavery

**Affiliations:** a Centre of Evidence Based Dermatology, University of Nottingham, Nottingham, UK; b Nuffield Department of Clinical Medicine, University of Oxford, Oxford, UK; c Institute of General Medicine and Interprofessional Care, University of Tübingen, Tübingen, Germany; d Warwick Medical School, University of Warwick, Coventry, UK; e Medical Research Council Clinical Trials Unit at University College London, London, UK; f Department of Dermatology, University of Lübeck, Lübeck, Germany; g Nottingham Clinical Trials Unit, Nottingham Health Science Partners, Nottingham, UK; h Dermatology Department, Hull Royal Infirmary, Hull, UK; i Dermatology Department, Leicester Royal Infirmary, Leicester, UK; j Dermatology Department, Queen Elizabeth Hospital, Greenwich, London, UK

## Abstract

**Background:**

Bullous pemphigoid is a blistering skin disorder with increased mortality. We tested whether a strategy of starting treatment with doxycycline gives acceptable short-term blister control while conferring long-term safety advantages over starting treatment with oral corticosteroids.

**Methods:**

We did a pragmatic, multicentre, parallel-group randomised controlled trial of adults with bullous pemphigoid (three or more blisters at two or more sites and linear basement membrane IgG or C3). Participants were randomly assigned to doxycycline (200 mg per day) or prednisolone (0·5 mg/kg per day) using random permuted blocks of randomly varying size, and stratified by baseline severity (3–9, 10–30, and >30 blisters for mild, moderate, and severe disease, respectively). Localised adjuvant potent topical corticosteroids (<30 g per week) were permitted during weeks 1–3. The non-inferiority primary effectiveness outcome was the proportion of participants with three or fewer blisters at 6 weeks. We assumed that doxycycline would be 25% less effective than corticosteroids with a 37% acceptable margin of non-inferiority. The primary safety outcome was the proportion with severe, life-threatening, or fatal (grade 3–5) treatment-related adverse events by 52 weeks. Analysis (modified intention to treat [mITT] for the superiority safety analysis and mITT and per protocol for non-inferiority effectiveness analysis) used a regression model adjusting for baseline disease severity, age, and Karnofsky score, with missing data imputed. The trial is registered at ISRCTN, number ISRCTN13704604.

**Findings:**

Between March 1, 2009, and Oct 31, 2013, 132 patients were randomly assigned to doxycycline and 121 to prednisolone from 54 UK and seven German dermatology centres. Mean age was 77·7 years (SD 9·7) and 173 (68%) of 253 patients had moderate-to-severe baseline disease. For those starting doxycycline, 83 (74%) of 112 patients had three or fewer blisters at 6 weeks compared with 92 (91%) of 101 patients on prednisolone, an adjusted difference of 18·6% (90% CI 11·1–26·1) favouring prednisolone (upper limit of 90% CI, 26·1%, within the predefined 37% margin). Related severe, life-threatening, and fatal events at 52 weeks were 18% (22 of 121) for those starting doxycycline and 36% (41 of 113) for prednisolone (mITT), an adjusted difference of 19·0% (95% CI 7·9–30·1), p=0·001.

**Interpretation:**

Starting patients on doxycycline is non-inferior to standard treatment with oral prednisolone for short-term blister control in bullous pemphigoid and significantly safer in the long-term.

**Funding:**

NIHR Health Technology Assessment Programme.

## Introduction

Bullous cutaneous pemphigoid is the most common autoimmune blistering skin disease characterised by autoantibodies against structural skin proteins of the dermal–epidermal junction.^1^ Annual incidence of bullous pemphigoid in the UK and central Europe ranges between 14 and 42 new patients per million inhabitants and has doubled within the last decade.^2^, ^3^ Bullous pemphigoid is an intensely itchy condition characterised by erythema studded with tense blisters, some of which might become infected. The disorder is commoner in those older than 70 years and runs a chronic progressive course.^4^ It is associated with increased morbidity and mortality,^2^, ^5^ neurological diseases including dementia,^6^, ^7^ Parkinson's disease, motor neurone disease and stroke,^6^, ^7^, ^8^ haematological malignancies,^9^ and exposure to some medications,^8^ such as loop diuretics.^10^ Oral prednisolone has been the standard treaWtment for bullous pemphigoid for more than 50 years,^4^, ^11^ but is associated with clinically significant adverse effects in the elderly and the optimal dose is uncertain. Prolonged whole body application of super-potent topical corticosteroids has been shown to be effective in bullous pemphigoid,^12^ with less morbidity and mortality than oral high-dose corticosteroid treatment.^3^, ^13^ However, this regimen might not be practical for patients with limited mobility and limited help from carers to apply whole body ointments daily. Systemic absorption when applied to the whole body might be considerable,^12^ so there remains a need for an oral treatment that is safe and effective. Tetracyclines have been used in bullous pemphigoid for their anti-inflammatory action,^14^, ^15^ but a Cochrane systematic review found only one small study supporting their use.^16^ It is unlikely that tetracyclines would be more effective than oral prednisolone, but they are expected to be safer in the long term. We did a survey of UK dermatologists that suggested most were willing to accept a 25% reduction in early blister control with doxycycline provided there was at least a 20% reduction in serious related side-effects compared with prednisolone. The Bullous Pemphigoid Steroids and Tetracyclines (BLISTER) study^17^ tested whether a strategy of starting treatment with tetracyclines produces an acceptable degree of short-term blister control compared with oral corticosteroids (a non-inferiority comparison), while conferring a long-term safety advantage over oral corticosteroids (a superiority comparison) in a pragmatic way that could inform everyday clinical practice.

**Research in context**

**Evidence before this study**

We updated a Cochrane systematic review of interventions for bullous pemphigoid in 2010 (no language restrictions) and found ten randomised controlled studies, only one of which (20 participants) tested use of tetracyclines—despite their widespread use. We searched the Cochrane Library and PubMed for publications from Aug 1, 2010, to Nov 1, 2016, using the term “pemphigoid” and did not find any new trials of tetracyclines for bullous pemphigoid. The most substantial advance in pemphigoid treatment in the last 10 years has been the demonstration of good response rates for superpotent and potent topical corticosteroids applied to the whole body for prolonged periods with substantial reduction in mortality when compared with oral prednisolone. Use of topical steroid treatment is partly limited by side-effects such as skin thinning and practical considerations such as reduced mobility or lack of carer support to enable elderly patients to apply whole body ointments daily, so there is still a need for an oral treatment for bullous pemphigoid that is safer than prednisolone.

**Added value of this study**

Our study provides new information that supports our hypothesis that starting patients with bullous pemphigoid on oral doxycycline 200 mg per day produces acceptable blister control in the short term (within our a-priori non-inferiority limits), with better long-term safety than starting on prednisolone 0·5 mg/kg per day. We did not find any evidence that the treatment response differed between the two strategies according to the severity of blisters at baseline. The relative trade-off of blister control versus long-term safety provides key information for shared decision making between patients with bullous pemphigoid and doctors.

**Interpretation of all the available evidence**

Topical corticosteroid treatment still probably offers the best trade-off between blister control and long-term safety when compared with oral prednisolone, but prolonged whole body topical treatment might not always be practical in some patients, such as the elderly. In individuals with bullous pemphigoid who need oral therapy, our study suggests that initiating treatment with doxycycline might produce acceptable blister control and better long-term safety than standard treatment with oral prednisolone.

## Methods

### Study design and participants

The study was a two-group parallel pragmatic multicentre randomised controlled clinical trial of 52 weeks duration done in dermatology specialist clinics in the UK and Germany.^18^, ^19^ The trial used a non-inferiority approach to compare short-term effectiveness and a superiority approach to compare long-term safety.^17^, ^18^

Adults (≥18 years of age) attending dermatology specialist clinics with suspected bullous pemphigoid and at least three clinically significant blisters appearing on at least two body sites within the last week and who were able to provide written informed consent were enrolled. Clinically significant blisters were defined as cutaneous blisters at least 5 mm in diameter, inclusive of ruptured blisters with a flexible (not dry) roof covering a moist base. All patients with suspected bullous pemphigoid had their clinical diagnosis confirmed by positive direct or indirect immunofluorescence (IgG or C3 at the dermal–epidermal junction or both) or were subsequently excluded if immunofluorescence was negative. Further exclusions were a diagnosis of mucous membrane pemphigoid; a documented diagnosis of active bullous cutaneous pemphigoid in the year before randomisation; use of study medications in the previous 12 weeks; recent (3 months or less) administration of a live virus vaccine; known allergy to any member of the tetracycline family; presence of any condition or use of any medication which precludes the use of either of the study drugs; women of childbearing potential who are not taking adequate contraception or who are pregnant, plan to become pregnant during the study duration or lactating; any other condition which would, in the investigator's opinion, deem the patient unsuitable for participation in the study (eg, condition requiring long-term or frequent oral steroid use); and patient participating in any other intervention study.

Trial oversight was by a Trial Steering Committee and an independent Data Monitoring Committee. Ethics permission was granted for the UK by Central Manchester Research Ethics Committee (Nov 12, 2008; reference 08/H1008/174) and for Germany by Ethik-Kommission der Universität zu Lübeck, Medizinische Fakultät (Aktenzeichen 09-134). The trial protocol and statistical analysis plan are available online.

### Randomisation and masking

Participants were randomly assigned (1:1) to receive either doxycycline or prednisolone as initial treatment, and followed up for skin and medication assessments at weeks 3, 6, 13, 26, 39, and 52, plus unscheduled visits as required, reflecting normal clinical care. Randomisation was done by the internet and occurred once recruited participants' details had been entered by local physicians and research nurses onto a study database. Treatment was allocated using random permuted blocks of randomly varying size generated by the Nottingham Clinical Trials Unit and was stratified by baseline severity (3–9, 10–30, and >30 blisters for mild, moderate, and severe disease, respectively). Treatment allocation was sent directly to the local pharmacist who dispensed the appropriate medication directly, allowing the investigator to remain masked for the first 6 weeks. Investigators were subsequently unmasked to adjust or switch medication to reflect normal clinical practice. Participants were not masked to study medications. Given the morbidity of the elderly population and the fact that the characteristic adverse events of each drug are very well described, only adverse events suspected to be related were recorded. Adverse event collection after 6 weeks was not masked. Relatedness of unclear serious adverse events and all deaths was judged by a senior dermatologist independent of the trial team.

### Procedures

We compared a strategy of initial treatment with oral doxycycline 200 mg per day or oral prednisolone 0·5 mg/kg per day for 6 weeks until the primary effectiveness outcome was measured, followed by a period in which the investigators were unmasked to treatments so that they could either continue with the randomised treatment if blister control was adequate, or switch treatments or adjust the dose of prednisolone, as might occur in normal clinical practice. Treatment with either strategy could continue up to 12 months according to blister control and adverse effects. We chose doxycycline because oxytetracycline requires participants to swallow eight large tablets daily and doxycycline has fewer adverse effects.^20^ We chose a dose of 0·5 mg/kg per day of prednisolone because a previous systematic review suggested higher doses were associated with unacceptable serious adverse effects^16^ and lower doses might be ineffective.^21^ Up to 30 g per week of a potent topical corticosteroid (mometasone furoate) was permitted to be applied to localised lesions for symptom relief for weeks 1–3 only, and again after 6 weeks as per normal practice.^15^ Only lesional and not whole body application of topical corticosteroids was permitted.

### Outcomes

We included two primary outcomes to measure the trade-off between effectiveness (initial blister control) and safety (serious adverse effects) for the two treatment strategies. Short-term control (effectiveness) was measured at 6 weeks after randomisation, and long-term safety was measured at 52 weeks after randomisation. The primary outcomes were the absolute difference between the treatment groups in: the proportion of participants classed as treatment success (three or fewer significant blisters) at 6 weeks, regardless of whether their treatment had been modified because of a poor response (non-inferiority effectiveness comparison of treatment strategy); and the proportion of participants with grade 3–5 (severe, life-threatening, or fatal) adverse events that were possibly, probably, or definitely related to the treatment in the 52 weeks following randomisation (superiority safety comparison). A modified version of the Common Terminology Criteria for Adverse Events (version 3.0) was used.

Secondary effectiveness outcomes were the proportion of participants who were deemed treatment successes (three or fewer significant blisters and no treatment modification before 6 weeks as a more direct comparison of drug rather than treatment strategy), the proportion classed as treatment success at 13 and 52 weeks (three or fewer significant blisters and no treatment modification), and relapses (those with further episodes of bullous pemphigoid during the study who had previously been classed as success). Secondary safety outcomes were the proportion reporting related adverse events of any grade up to week 52, participants classed as a treatment success at 6 weeks still alive at 52 weeks, quality-of-life (EuroQoL EQ-5D-3L and Dermatology Life Quality Index [DLQI] questionnaires at 6, 13, 26, 39, and 52 weeks), and cost-effectiveness over 12 months from a UK health service perspective. The cost-effectiveness analysis will be published in detail elsewhere.

Tertiary outcomes were the proportion of participants who were deemed treatment successes (three or fewer significant blisters and no treatment modification) at 3 weeks, proportion of patients completely blister free by 6 weeks, all-cause mortality at 52 weeks, and the amount of localised use of potent and super-potent topical corticosteroids (as recorded in the treatment log by local physicians).

### Statistical analysis

For the safety primary endpoint, 256 patients were needed to show a reduction from 60% to 40% in adverse reactions of grade 3 or more for those started on doxycycline compared with prednisolone, with 80% power, 5% significance, and allowing for 20% loss to follow-up at 1 year.^13^, ^22^ When estimating the number of participants required for the effectiveness analysis we assumed that doxycycline would be less effective than prednisolone, with around 70% having three or fewer blisters at 6 weeks compared with 95% in the prednisolone-initiated group, an absolute difference of 25%, based on published data and expert opinion. We set the acceptable non-inferiority margin at 37%, corresponding to the upper bound of the 90% CI for the anticipated 25% difference in effectiveness. This required a total of 234 participants to show non-inferiority with 80% power, allowing for a 5% expected dropout by 6 weeks. For initial treatment with doxycycline to be considered an acceptable alternative strategy to prednisolone, both superiority for safety and non-inferiority for effectiveness had to be shown. Because bullous pemphigoid is rare and mainly seen in the very elderly, we were restricted in the number of participants we could realistically expect to recruit. The smaller the non-inferiority margin, the greater the sample size, so to set the margin closer to the expected 25% difference in effectiveness would have required an unrealistically large sample size. Therefore, the non-inferiority margin was set at 37% (the 25% expected difference plus a further 12%) which meant that 234 participants would be sufficient. Thus 37% was the maximum allowable difference for a strategy of starting treatment with doxycycline to be considered non-inferior. Given these considerations and the sample size required for the safety analysis it was decided to recruit a total of 256 patients. For initial treatment with doxycycline to be considered an acceptable alternative strategy to prednisolone, non-inferiority had to be shown for effectiveness and superiority for safety.

Analysis used a binomial regression model with an identity link function adjusting for baseline severity of bullous pemphigoid, patient age, and Karnofsky score for functional impairment to estimate the absolute difference between the two treatment groups, and missing data were imputed. Superiority analyses were done on a modified intention-to-treat basis (participants who fulfilled eligibility were randomly assigned to either of the study drugs and had data on the outcome of interest as pre-defined in our protocol) and non-inferiority analyses were done on both modified intention-to-treat and per-protocol populations according to recommended practice^23^ and according to the protocol and statistical analysis plan. The per-protocol population for the non-inferiority outcomes at weeks 3 and 6 was defined as those participants who for reasons other than treatment success or failure (determined by the investigators) had not increased their dose of allocated treatment, changed treatment or added a new treatment to their allocated treatment, used topical steroids between visit weeks 3 and 6 (week 6 outcome only), missed more than 3 consecutive treatment days, or committed other deviations deemed to be violations by an independent adjudicator masked to treatment allocation. After week 6, the per-protocol population for non-inferiority analyses was those included in 6-week primary effectiveness per-protocol analysis who had not missed more than 3 consecutive weeks of allocated treatment between 6 weeks and 52 weeks (regardless of whether their dose had been increased or decreased) unless they had stopped for good clinical response, those who had not used more than 30 g of potent topical steroids per week after week 6, had not added systemic steroids to doxycycline (if allocated) or doxycycline or another immunosuppressant to prednisolone (if allocated) unless for poor clinical response, or did not commit other protocol deviations deemed as violations by an independent adjudicator.

Multiple imputation methods for dealing with missing data for the primary safety analyses can be found in the statistical analysis plan. Two planned subgroup analyses for treatment success at 6 weeks explored interactions with baseline disease severity and instances where investigators had become unmasked to treatment allocation before week 6.

There were no substantial changes to the main study protocol after recruitment started. The trial is registered at ISRCTN, number ISRCTN13704604.

### Role of the funding source

The funder of the study had no role in study design, data collection, data analysis, data interpretation, writing of the report, or decision to submit the results for publication. The corresponding author and trial statisticians had full access to all the data in the study. All authors in the writing team shared final responsibility for the decision to submit for publication. The full report submitted to the funder is available elsewhere.^17^

## Results

**Figure 1:**
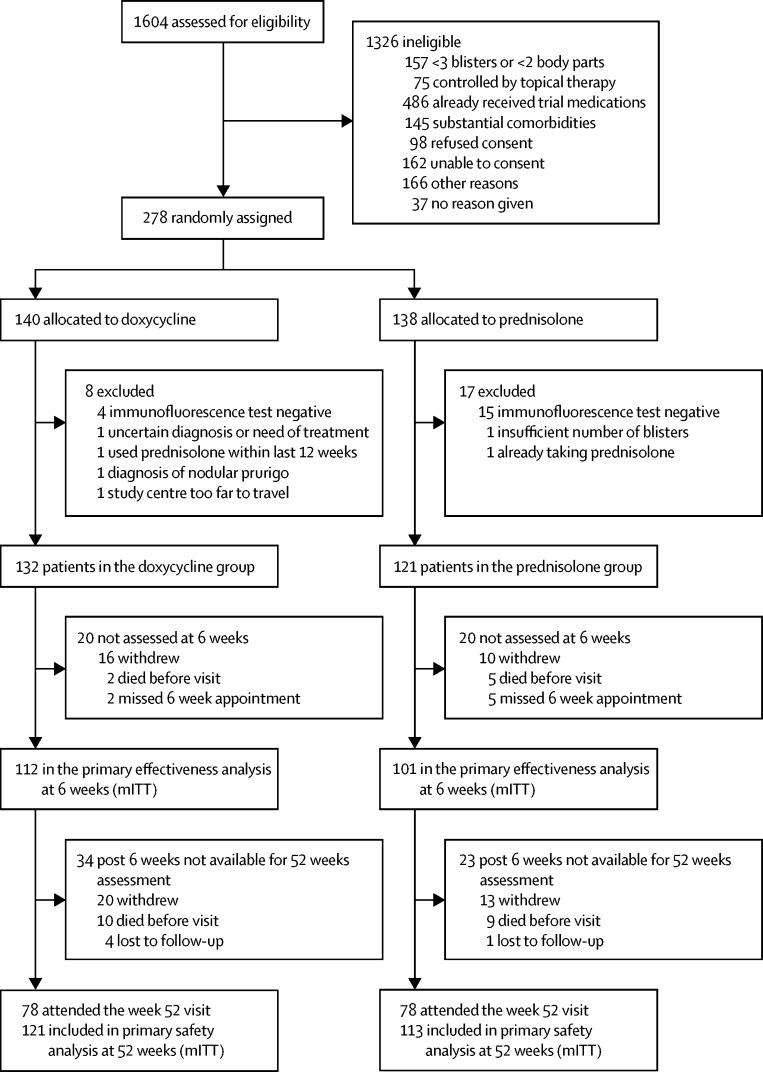
Trial profile for the primary outcome population Those patients excluded from analysis at week 6 due to missing their week 6 assessment are included in the denominator at week 52, as they had the possibility of attending a visit after week 6 and therefore were not considered lost to follow-up at week 6. Patients who did not attend their week 52 visit are called lost to follow-up at week 52. The primary safety analysis (mITT) was based on 121 and 113 participants allocated to initial doxycycline and prednisolone, respectively, who had at least one return visit. Multiple imputation was used for missed visits as specified in the protocol. mITT=modified intention to treat.

We randomly allocated 278 participants from dermatology specialist clinics in 54 UK and seven German centres. Recruitment commenced on March 1, 2009, in the UK and Feb 1, 2010, in Germany, and ended on Oct 31, 2013. 19 participants were excluded because their immunofluorescence tests were negative and six for other reasons; 253 participants (132 in the doxycycline group and 121 in the prednisolone group) were available for analysis (figure 1). Baseline characteristics of the study population in both treatment groups were similar (table 1). Average age was 77·7 years (SD 9·7); 80 (32%) participants had mild disease, 99 (39%) had moderate disease, and 74 (29%) had severe disease. The number and primary reasons for withdrawal from the trial are in the appendix.

**Figure 2:**
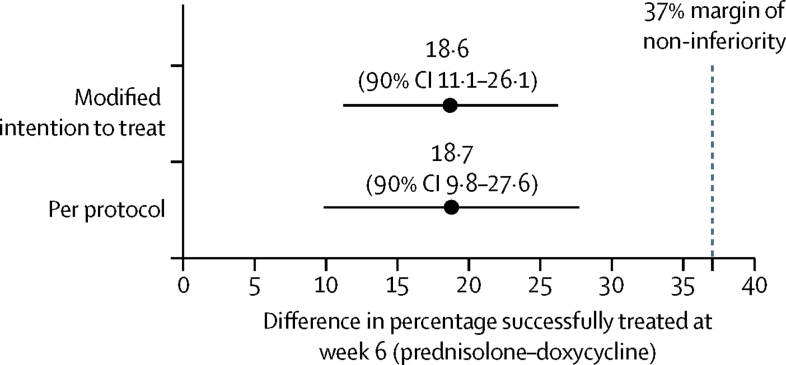
Proportion of participants who achieved treatment success at 6 weeks: the modified intention-to-treat and per-protocol analyses

For the primary outcome, the number of participants who achieved success (three or fewer significant blisters) with doxycycline-initiated treatment was 83 (74%) of 112 patients compared with 92 (91%) of 101 patients started on prednisolone, an adjusted difference of 18·6% (90% CI 11·1–26·1; table 2). The upper bound CI of 26·1% for the adjusted difference fell within the 37% pre-specified acceptable margin for the 90% CI. This value remained close regardless of whether a modified intention-to-treat or per-protocol analysis was used or whether adjusted by severity and Karnofsky score as shown in figure 2.

Subgroup analysis of the primary outcome according to baseline severity shows that the benefit of both treatments diminishes in severe disease, although there was no significant interaction between treatment effect and severity (p_interaction_=0·874 for moderate *vs* mild baseline severity and p_interaction_=0·431 for severe *vs* mild baseline severity; table 2). Subgroup analysis of the primary outcome according to whether masking was compromised did not show any evidence of interaction for modified intention-to-treat (p=0·333) or per-protocol analyses (p=0·356).

For the primary safety outcome, the number of patients experiencing a treatment-related severe, life-threatening, or fatal adverse event by 52 weeks was 22 (18%) of 121 for patients started on doxycycline compared with 41 (36%) of 113 for those starting on prednisolone, a difference of 18·1% (95% CI 6·9–29·3, p=0·002) when unadjusted, and 19·0% (7·9–30·1, p=0·001) after adjusting for baseline disease severity (table 3). Similar results were seen with imputed missing values (table 3). There were three treatment-related deaths in the doxycycline-initiated group compared with 11 in the prednisolone-initiated group. The total number of related adverse events and maximum grade of related adverse events (≥grade 3) are shown in the appendix (pp 2, 3).

The proportion of patients achieving treatment success defined as three or fewer significant blisters and no treatment modification before 6 weeks in the modified intention-to-treat population was 60 (54%) of 112 in the doxycycline group and 88 (85%) of 103 in the prednisolone group (difference in favour of prednisolone 31·8% [90% CI 22·5–41·2] after adjusting for baseline disease severity and age). The proportion achieving treatment success without treatment modification (modified intention-to-treat population) at 3 weeks was 67 (56%) of 119 patients in the doxycycline group compared with 90 (81%) of 111 patients in the prednisolone group (difference 23·4% [90% CI 14·4–32·5] after adjusting for Karnofsky score). At 13 weeks, the numbers were 58 (59%) of 99 patients in the doxycycline group and 76 (75%) of 101 patients in the prednisolone group (adjusted difference 17·5% [90% CI 6·8–28·2]), and at 52 weeks, 34 (41%) of 83 patients in the doxycycline group and 45 (51%) of 88 patients in the prednisolone group (adjusted difference 10·0% [90% CI −2·3 to 22·2]).

Relapse rates (more than three blisters for those initially classed as treatment success at 6 weeks) were similar in both groups: 37 (32%) of 114 patients in the doxycycline group and 39 (36%) of 109 patients in the prednisolone group, an adjusted difference of 2·1% (90% CI −8·3 to 12·5; appendix p 4).

Patients in the prednisolone group were more likely to be completely blister free at 6 weeks than those in the doxycycline group (74 [73%] of 101 patients *vs* 51 [46%] of 111 patients, an adjusted difference of 28·6% (90% CI 18·1–39·1). Topical corticosteroid use for symptomatic relief was greater for those initiated on doxycycline treatment (appendix p 4).

Patients in the prednisolone group were significantly more likely to experience treatment-related adverse events of any grade during the study than those in the doxycycline group (96% *vs* 86%, difference 9·5% [95% CI 1·8–17·2], p=0·016, unadjusted because of non-convergence in the model).

The total number of related adverse events (all grades) and maximum grade are shown in the appendix (p 5). Safety analysis of all-cause mortality showed that patients started on prednisolone were more likely to die during the year than those who started on doxycycline (101 [83%] of 121 patients *vs* 118 [89%] of 132 patients alive at 1 year; appendix pp 6, 7). The Kaplan Meier survival plot of time to death is in the appendix (p 8).

The combined superiority analysis of effectiveness and safety (treatment success at 6 weeks and alive at 52 weeks) showed a difference of 25·0% (95% CI 13·1–37·0) in favour of prednisolone after adjusting for baseline disease severity and age (77 [75%] of 103 patients in the prednisolone group *vs* 56 [50%] of 112 patients in the doxycycline group).

For quality of life assessed by EQ-5D the difference in score was not significant when adjusted for baseline score, baseline disease severity, age, or Karnofsky score (adjusted difference 0·045 [95% CI −0·015 to 0·106], p=0·143; appendix p 9).

Both groups experienced similar improvement in DLQI scores with median improvement of 9 and 10 points from baseline in the doxycycline and prednisolone groups, respectively. When adjusted for baseline DLQI, disease severity, age, and Karnofsky score, there was a small but significant difference of −1·8 (95% CI −2·58 to −1·01, p<0·0001) in favour of doxycycline (appendix p 10).

Because three or more significant blisters was an inclusion criterion and treatment success was defined as three or fewer blisters, it is possible that some participants in the mild group could have experienced little or no change in blister status. No worsening in these patients at 6 weeks is a good result. However, when those in the mild severity group who had one to three blisters at 6 weeks (14 in the doxycycline group and seven in the prednisolone group) were re-categorised as no change in a post-hoc sensitivity analysis, there was no change in the magnitude or direction of the primary effectiveness outcome (85 [90%] of 94 patients had treatment success on prednisolone and 69 [70%] of 98 patients on doxycycline, a difference of 20·0% [90% CI 10·9–29·1%], in an adjusted modified intention-to-treat analysis analysis).

## Discussion

We have shown that a strategy of starting treatment for bullous pemphigoid with doxycycline 200 mg daily produces acceptable short-term effectiveness that resides within our predefined non-inferiority margin, and significant safety gains at 1 year compared with initiating treatment with prednisolone 0·5 mg/kg per day. Although we did not find clear evidence that differences between the two treatment strategies varied by baseline disease severity, our study suggests that effectiveness of either strategy is modest for those with severe disease.

Although previous studies^3^, ^13^ have shown good blister control and better safety for prolonged use of super-potent topical corticosteroids over the whole body when compared with high-dose oral prednisolone (mg/kg per day), such a regimen might be impractical for some patients and carers.^24^ Prolonged topical corticosteroid use also causes adverse effects such as skin thinning, and those effects associated with systemic absorption.^3^, ^13^ Most guidelines still recommend oral prednisolone (between 0·5 mg/kg per day and 1·5 mg/kg per day according to initial severity).^12^, ^25^ Most guidelines mention the widespread use of tetracyclines in bullous pemphigoid, but cite the lack of high-quality evidence to inform this treatment recommendation. The Cochrane review^16^ of bullous pemphigoid found only one randomised study of 18 patients treated with tetracycline and nicotinamide versus prednisolone, which had inconclusive results;^26^ none have been published since. The BLISTER study fills this gap.

Study strengths include the relatively large sample size and pragmatic design that permitted dose adjustment, switching, and additional treatments as per normal clinical practice. The perspective of the trial was one of a long-term treatment strategy of starting patients with bullous pemphigoid on doxycycline versus prednisolone rather than a direct efficacy study comparing sole use of each drug. If we had used our secondary outcome (proportion achieving treatment success at 6 weeks defined as three or fewer significant blisters but no treatment modification before 6 weeks), then the doxycycline group would have narrowly failed to achieve non-inferiority according to our predefined margins.

It is unlikely that the localised use of small quantities of topical corticosteroids for symptomatic relief permitted in the first 3 weeks of treatment affected effectiveness comparisons at 6 weeks. The nature of such localised topical treatment for symptom relief is very different from the whole-body application of large quantities of super-potent or potent topical corticosteroids used in other studies.^3^, ^13^ Per-protocol analyses that excluded participants who used topical corticosteroids in weeks 4–6 showed very similar results to the modified intention-to-treat analysis in which they were included.

Participants were representative of those presenting to secondary care from multiple centres in the UK and Germany. Indirect immunofluorescence on human split-skin was not done at all centres, so it is possible that a small number of participants with epidermolysis bullosa acquisita were included. However, epidermolysis bullosa acquisita accounts for less than 5% of pemphigoid disorders, is often unreactive by indirect immunofluorescence microscopy on monkey oesophagus, and any cases would have been randomly distributed between the treatment groups. Furthermore, none of the study centres reported that the diagnosis of pemphigoid was revised to another disease during the 1 year of follow-up. It is also worth emphasising that any potential participants who did not show a clear direct or indirect positive immunofluorescence were excluded from the study and were replaced by other participants with clear evidence of bullous pemphigoid. Masking of outcome assessment in the first 6 weeks was largely maintained. Exclusion of participants with dementia—because we required patients to give fully informed consent (in line with the ethics committee guidance)—means our study results might not be applicable to those with non-bullous cutaneous pemphigoid (pre-pemphigoid). Placebo effect or regression to the mean is an unlikely explanation for the observed responses for doxycycline, given the progressive nature of bullous pemphigoid. Although no consideration of reverse multiplicity was made at the time the sample size was determined, the analyses of the primary effectiveness endpoint showed that the upper confidence difference for the effectiveness of the doxycycline group, 26·1%, was well below 37%, the chosen margin of non-inferiority, and the benefits in terms of safety were significant.^27^ The similarity of the inclusion criterion of three or more significant blisters to the primary success criterion of three or fewer blisters meant it was possible that some participants in the mild disease group experienced little change in blister status, although a sensitivity analysis was robust when excluding such participants from the analysis. Some degree of attribution bias could have occurred by wrongly assigning some unrelated serious adverse events as related ones, on the basis of knowledge of the side-effects of the study drugs. To reduce this, all unclear grade 3–5 events were reviewed by a senior independent dermatologist. It is unlikely that the safety outcomes at 52 weeks could be attributed to other drugs used after week 6. Similar proportions of patients received azathioprine (4% [four of 113 patients] in the prednisolone group and 8% [ten of 121 patients] in the doxycycline group) and no serious adverse drug reactions were attributed to azathioprine. Although bullous pemphigoid is characterised by a progressive course, the kinetics of its progression varies among patients, especially in patients with mild-to-moderate disease. We failed to show any clear differences between mild, moderate, and severe disease in the short, medium, or long term, but it is possible that such differences could be shown in a much larger study or in an efficacy study done solely in patients with mild disease. Some of the participants in this study with milder or less progressive disease could have benefited from topical treatments. Dropouts for long-term outcomes were quite high, as anticipated in this frail elderly population, but were mostly due to withdrawal of consent and death rather than lack of effectiveness or adverse events (appendix p 2) and primary outcomes remained stable when adjusting for missing data for using multiple imputation.

The upper limit of the 90% CI around the difference of 18·6% between the two treatment strategies (adjusted modified intention-to-treat analysis) is compatible with a difference as small as 26·1%, which falls well within the pre-determined 37% non-inferiority margin and is very close to the level of 25% in which a sample of dermatologists, when surveyed before the study (appendix p 1),^17^ said would be acceptable provided it was accompanied by a clinically important reduction in side-effects.

Definitions and outcomes for future trials in bullous pemphigoid proposed in 2015 (6 years after our study was designed) include 13 different outcome definitions according to the period of observation.^28^ Some might think that the absence of recording pruritus, which can herald the onset and recurrence of bullous pemphigoid, was a limitation of this study. However, it was always our intention to keep participant questionnaire burden to an absolute minimum in this pragmatic study in an elderly population, and quality-of-life measurements might have reflected symptoms such as itching, as well as other symptoms such as pain, burning, and soreness.

Because short-term control of blisters is less than optimal with doxycycline, future research might evaluate a strategy of starting all patients with a short course of super-potent topical corticosteroids or prednisolone 0·5 mg/kg per day, followed by randomisation to maintenance treatment with either oral doxycycline or continuation with corticosteroids or other immunsuppressive maintenance treatments such as weekly oral methotrexate. Future studies should also consider including patients with bullous pemphigoid who have dementia, given the association of bullous pemphigoid with neurological diseases and the suggestion that tetracyclines might have neuroprotective properties.^29^, ^30^ Although good progress has been made in identifying possible standardised outcomes such as the Bullous Pemphigoid Disease Area Index and other definitions for use in bullous pemphigoid trials,^28^ a minimum set of core outcome measures are still needed.

This study provides new evidence that doxycycline has a positive effect in controlling bullous pemphigoid blisters when used for initial treatment as part of a long-term treatment strategy. The study confirms that oral prednisolone at a dose of 0·5 mg/kg per day is highly effective for mild-to-moderate disease, albeit at the expense of potentially serious, life-threatening, and fatal related adverse events compared with doxycycline. Both drugs are relatively cheap and available worldwide.

Where whole-body application of super-potent topical corticosteroids for months is not practical, a strategy of starting oral doxycycline plus localised application of potent topical corticosteroids to blisters^15^ might be considered in preference to the current standard practice of starting such patients on oral prednisolone. The estimates of the trade-off between reduced short-term effectiveness versus long-term safety gains according to disease severity obtained from this study now provide clear data to inform shared treatment decisions between health-care professionals and patients with bullous pemphigoid or their carers.

For more on the **trial protocol and statistics analysis plan** see http://www.nottingham.ac.uk/research/groups/cebd/projects/5rareandother/index.aspx

For more on the **UK Dermatology Clinical Trials Network** see www.ukdctn.org

## Figures and Tables

**Table 1:** Baseline characteristics of trial participants

		**Doxycycline (n=132)**	**Prednisolone (n=121)**	**Total (n=253)**
Sex				
	Female	63 (48%)	57 (47%)	120 (47%)
	Male	69 (52%)	64 (53%)	133 (53%)
Age (years)		78·1 (9·5)	77·2 (10·0)	77·7 (9·7)
	<65	8 (6%)	14 (12%)	22 (9%)
	65–74	38 (29%)	33 (27%)	71 (28%)
	75–84	51 (39%)	45 (37%)	96 (38%)
	≥85	35 (27%)	29 (24%)	64 (25%)
Karnofsky score		69·0 (18·3)	70·5 (18·6)	69·7 (18·0)
	<40	3 (2%)	1 (1%)	4 (2%)
	40–54	32 (24%)	26 (21%)	58 (23%)
	55–69	21 (16%)	24 (20%)	45 (18%)
	70–84	45 (34%)	38 (31%)	83 (33%)
	≥85	31 (23%)	32 (26%)	63 (25%)
Unable to care for self		16 (12%)	11 (9%)	27 (11%)
Unable to work		55 (42%)	51 (42%)	106 (42%)
Able*		61 (46%)	59 (49%)	120 (47%)
Ethnicity				
	White	112 (85%)	100 (83%)	212 (84%)
	Black African	1 (1%)	1 (1%)	2 (1%)
	Black other	0	1 (1%)	1 (<1%)
	Asian Indian	2 (2%)	1 (1%)	3 (1%)
	Asian Chinese	1 (1%)	0	1 (<1%)
	Asian other	2 (1·5%)	1 (1%)	3 (1%)
	Other	0	1 (1%)	1 (<1%)
	Not known or not given	14 (11%)	16 (13%)	30 (12%)
Severity of bullous pemphigoid				
	Mild (3–9 blisters)	42 (32%)	38 (31%)	80 (32%)
	Moderate (10–30 blisters)	53 (40%)	46 (38%)	99 (39%)
	Severe (>30 blisters)	37 (28%)	37 (31%)	74 (29%)

Data are n (%) or mean (SD).

* Able to carry on normal activity and to work; no additional care needed.

**Table 2:** Proportion of participants who achieved treatment success (three or fewer blisters) at 6 weeks by modified intention-to-treat and per-protocol analyses, and subgroup analysis of baseline disease severity and treatment effect by modified intention-to-treat analysis

	mITT population*(all severities†**)**	**Per-protocol population**‡	mITT population*		
			Mild disease†	Moderate disease†	Severe disease†
Prednisolone (n/N, %)	92/101 (91%)	84/91 (92%)	30/31 (97%)	41/42 (98%)	21/28 (75%)
Doxycycline (n/N, %)	83/112 (74%)	58/78 (74%)	28/37 (76%)	36/46 (78%)	19/29 (66%)
Adjusted difference in proportions (%, 90% CI)	18·6% (11·1 to 26·1)§	18·7% (9·8 to 27·6)§	21·1% (7·7 to 34·5)¶	19·3% (7·7 to 31·0)¶	9·5% (−10·4 to 29·4)¶
Unadjusted difference in proportions (%, 90% CI)	17·0% (8·7 to 25·2)	17·9% (8·6 to 27·3)	21·1% (8·4 to 33·8)	19·4% (8·6 to 30·1)	9·5% (−10·3 to 29·3)
p_interaction_‖	..	..	..	0·874	0·431

For doxycycline to be considered non-inferior to the control treatment, the upper bound of the 90% CI should fall below 37%. mITT=modified intention to treat.

* All randomised patients who had a blister count at 6 weeks.

† Baseline severity; mild=<10 blisters at baseline; moderate=10–30 blisters at baseline; severe=>30 blisters at baseline.

‡ Those participants who before their 6 week visit had not increased the dose of their allocated treatment, changed treatment, or added a new treatment to their allocated treatment (for a reason other than for treatment failure or success), used topical steroids between visit weeks 3 and 6, or missed more than 3 consecutive days of treatment.

§ Estimates are from a regression model adjusted for baseline severity of bullous pemphigoid and Karnofsky score; age was omitted from the model due to model non-convergence when present.

¶ Estimates are from a regression model containing an interaction between treatment group and baseline severity of disease, also adjusted for Karnofsky score; age was omitted from the model due to model non-convergence when present.

‖ A post-hoc test for interaction was done comparing the mild disease group with both the moderate and the severe disease groups for the adjusted model; a global interaction test gave a p_interaction_ of 0·709.

**Table 3:** Proportion of participants experiencing at least one grade 3–5 adverse event by 52 weeks that was possibly, probably, or definitely related to study treatment using a modified intention-to-treat analysis for a model based on the raw dataset and imputed dataset

	**Raw dataset**	**Imputed dataset**
Prednisolone (n/N, %)	41/113 (36%)	40·0%*
Doxycycline (n/N, %)	22/121 (18%)	22·5%*
Adjusted† difference in proportions (%, 95% CI)	19·0% (7·9–30·1); p=0·001	18·4% (6·0–30·8); p=0·004
Unadjusted difference in proportions (%, 95% CI)	18·1% (6·9–29·3); p=0·002	17·5% (4·8–30·1); p=0·007

* Estimates from unadjusted regression model on imputed dataset.

† Estimates are from a regression model adjusted for baseline severity of bullous pemphigoid; however, Karnofsky score and age were omitted from the model due to model non-convergence with it present.
