# Supplementary material for: Doxycycline versus prednisolone as an initial treatment strategy for bullous pemphigoid: a pragmatic, non-inferiority, randomised controlled trial
Source: Lancet. 2017 Apr 22;389(10079):1630–8. doi: 10.1016/S0140-6736(17)30560-3 (PMC5400809; doi:10.1016/S0140-6736(17)30560-3)
Supplement: Supplementary appendix [file mmc1.pdf]

THE LANCET

Supplementary appendix

This appendix formed part of the original submission and has been peer reviewed. We post it as supplied by the authors.

Supplement to: Williams HC, Wojnarowska F, Kirtschig G, et al, on behalf of the UK Dermatology Clinical Trials Network BLISTER Study Group. Doxycycline versus prednisolone as an initial treatment strategy for bullous pemphigoid: a pragmatic, non-inferiority, randomised controlled trial. *Lancet* 2017; published online March 6. http://dx.doi.org/10.1016/S0140-6736(17)30560-3.

SUPPLEMENTARY APPENDIX

Table 1: Results of UKDCTN survey

**Question 1: What reduction in effectiveness would be acceptable?**
**(a) Assuming the *mortality rate* with oxytetracycline is *1% less* than with prednisolone and prednisolone is 95% effective:**
**Results:**<br>o a minimum median effectiveness rate of *50%* would be required for oxytetracycline to have *any place* in the management of BP<br>o a minimum median effectiveness rate of *80%* would be required oxytetracycline to have *potential* as primary treatment for BP.
**(b) Assuming the *mortality rate* with oxytetracycline is *10% less* than with prednisolone and prednisolone is 95% effective:**
**Results:**<br>o a minimum median effectiveness rate of **40%** would be required for oxytetracycline to have **any place** in the management of BP<br>o a minimum median effectiveness rate of **70%** would be required oxytetracycline to have **potential** as primary treatment for BP.
**Question 2: What reduction in mortality would be useful?**
**(a) Assuming oxytetracycline is *5% less effective* than prednisolone and the mortality rate of prednisolone is 40%:**
**Results:**<br>o a maximum median mortality rate of *35%* would be acceptable for oxytetracycline to have *any place* in the management of BP<br>o a maximum median mortality rate of *23%* would be acceptable for oxytetracycline to have *potential* as primary treatment for BP.
**(b) Assuming oxytetracycline is *10% less effective* than prednisolone and the mortality rate of prednisolone is 40%:**
**Results:**<br>o a maximum median mortality rate of *30%* would be acceptable for oxytetracycline to have *any place* in the management of BP<br>o a maximum median mortality rate of *20%* would be acceptable for oxytetracycline to have *potential* as primary treatment for BP.
**(c) Assuming oxytetracycline is *20% less effective* than prednisolone and the mortality rate of prednisolone is 40%:**
**Results:**<br>o a maximum median mortality rate of *30%* would be acceptable for oxytetracycline to have *any place* in the management of BP<br>o a maximum median mortality rate of *15%* would be acceptable for oxytetracycline to have *potential* as primary treatment for BP.

Table 2: Number of participants withdrawn from the trial and the primary reasons

Primary reason for withdrawal	Doxycycline	Prednisolone	Total
Death	14	19	33*
Adverse event	2	1	3
Lost to follow-up	5	4	9
Treatment failure	4	1	5
Withdrew consent	23	16	39
Unable to tolerate trial medications	1	0	1
Other	1	1	2
**Total**	**50**	**42**	**92**

*34 deaths occurred in the trial, but one participant withdrew consent before they subsequently died, hence the total withdrawn due to death is 33

Table 3: Total number of Grade 3, 4 and 5 related adverse events: mITT analysis

	Treatment	
	Doxycycline (n=121)	Prednisolone (n=113)
Total number of grade 3 (severe) AEs reported (mean per participant)	33* (0·3)	59 (0·5)
Total number of grade 4 (life-threatening) AEs reported (mean per participant)	9 (0·1)	9 (0·1)
Total number of grade 5 (death) AEs reported (mean per participant)	3 (<0·1)	11 (0·1)
**Total number of grade 3, 4 and 5 AEs reported** (mean per patient)	**45 (0·4)**	**79 (0·7)**

*includes one Suspected Unexpected Serious Adverse Reaction (hypoglycaemia)

Table 4: The maximum grade of adverse event that each participant experienced during the trial that was considered possibly, probably or definitely related to the study drug: mITT analysis

**The maximum grade of AE that each participant experienced during the trial that was considered possibly, probably or definitely related to study drug·**	**Treatment**	
	**Starting on Doxycycline (n=121)**	**Starting on Prednisolone (n=113)**
	**n (%)**	**n (%)**
Number of participants who either experienced no adverse events or whose maximum grade of AE was grade 1 (mild) or 2 (moderate)	99 (81·8%)	72 (63·7%)
Number of participants whose maximum grade of AE was grade 3 (severe)	14 (11·6%)	25 (22·1%)
Number of participants whose maximum grade of AE was grade 4 (life-threatening)	5 (4·1%)	5 (4·4%)
Number of participants whose maximum grade of AE was grade 5 (death)	3 (2·5%)	11 (9·7%)
**Total number of participants whose maximum grade of AE was 3, 4, or 5**	**22 (18·2%)**	**41 (36·3%)**

Note:· This table includes all patients who have at least 1 record of an AE assessment from the scheduled visit AE assessments, or a recorded SAE, SAR, or death. Participants count only once in this table as per their highest grade of adverse event.·

Table 5: Proportion of participants who had a further episode of BP after previously being classified as a treatment success – modified ITT analysis

	Treatment	
**Outcome**	**Doxycycline**	**Prednisolone**
Relapse	37 (32·5%)	39 (35·8%)
No relapse	77 (67·5%)	70 (64·2%)
**Total**	**114**	**109**
**Difference in proportions (prednisolone minus doxycycline)**	Adjusted*	**2·1%** (90% CI: -8·3% to 12·5%)
	Unadjusted	**3·3%** (90% CI: -7·1% to 13·8%)

Note:· All patients who have at least had one physical examination in the study, and who have been classed as having been classified as a treatment success at some point in the trial are included in this analysis·*Estimates are from a regression model adjusted for baseline severity of BP, age, and Karnofsky score·

Table 6: Potent and superpotent topical corticosteroid use according to study period and treatment allocation

	Doxycycline (N = 112), n (%)			Prednisolone (N = 101), n (%)		
**Time period**	**Potent**	**Superpotent**	**Patients using topical corticosteroids**	**Potent**	**Superpotent**	**Patients using topical corticosteroids**
After randomisation up to 3 weeks	6 (5.4)	1 (0.9)	7 (6.3)	3 (3.0)	1 (1.0)	4 (4.0)
After 3 weeks up to 6 weeks	12 (10.7)	11 (9.8)	23 (20.5)	6 (5.9)	0	6 (5.9)
After 6 weeks	5 (4.5)	7 (6.3)	12 (10.7)	2 (2.0)	0	2 (2.0)
At any time during the trial	13 (11.6)	11 (9.8)	24 (21.4)	6 (5.9)	1 (1.0)	7 (6.9)

Table 7: Total number of related adverse events by grade: raw data

Adverse event	Number of adverse events (mean per participant)	
	Doxycycline (*n* = 121)	Prednisolone (*n* = 113)
Grade 1 (mild)	210 (1.7)	234 (2.1)
Grade 2 (moderate)	158 (1.3)	129 (1.1)
Grade 3 (severe)	33 (0.3)	59 (0.5)
Grade 4 (life-threatening)	9 (0.1)	9 (0.1)
Grade 5 (death)	3 (< 0.1)	11 (0.1)
Grades 1–5	413 (3.4)	442 (3.9)

Note: This table includes all patients who have at least one record of an adverse event assessment from the scheduled visit adverse event assessments or a recorded serious adverse event or death.

Table 8: The maximum grade of adverse event that each participant experienced during the trial that was considered possibly, probably or definitely related to the study drug (any grade): raw data

Adverse event	Number (%) of patients	
	Doxycycline (*N* = 121)	Prednisolone (*N* = 113)
No adverse events	23 (19.0)	13 (11.5)
Maximum grade of adverse event grade 1 (mild)	20 (16.5)	16 (14.2)
Maximum grade of adverse event grade 2 (moderate)	56 (46.3)	43 (38.1)
Maximum grade of adverse event grade 3 (severe)	14 (11.6)	25 (22.1)
Maximum grade of adverse event grade 4 (life-threatening)	5 (4.1)	5 (4.4)
Maximum grade of adverse event grade 5 (death)	3 (2.5)	11 (9.7)
Adverse event of *any* grade	98 (81.0)	100 (88.5)

Note: This table includes all patients with at least one record of an adverse event assessment from the scheduled visit adverse event assessments or a recorded serious adverse event or death.

Table 9: All-cause mortality and reasons

Treatment allocated: Doxycycline			
**Patient number**	**Cause of death**	**Date of death**	**Possibly, probably or definitely related to trial treatment?**
5511	Sepsis secondary to Urinary tract Infection. Admitted with 24hr history of shivering and vomiting, temp on admission 38.4c 8.3. Treated for urosepsis with iv antibiotics and iv hydration. Prognosis poor, deteriorated, died 10/10/2012.	28/02/2012	Related
5512	Chest infection. Conditions contributing to the cause of death was stroke, hypertension and Bullous Pemphigoid. Patient switched treatments.	07/06/2012	Related
7101	Hypertension & severe LVF	05/06/2013	Unrelated
8407	died in hospital - MI & IHD	18/06/2012	Unknown
9604	Complication form pacemaker surgery. Follow-up information - medical notes now unobtainable. Will follow-up with HSCIC.	30/07/2009	Unknown
10103	1a.sepsis ,1b. Osteomyelitis of left foot. Contributing factor Diabetese mellitus and BP	06/10/2011	Unrelated
10605	Pneumonia, date of death 24/03/2012	05/01/2012	Unrelated
12104	Pulmonory emobolus, acute renal failure	14/12/2010	Unrelated
12302	1a congestivecardiac failure, 1b ischaemic heart disease. 2. COPD, 3. Morbid obesity	22/05/2009	Unrelated
12312	End of life pathway instigated by GP. Progressive frailty. Exact cause of death not known	19/12/2011	Unknown
12902	Pneumonia also cellulitis, also had COPD. BP in remission at time of death.	10/07/2012	Related
12903	Severe right heart failure and mitral stenosis. Died on ITU (on IV hydrocortisone at this point and not oral pred) after a prolonged hospital stay.	10/07/2012	Unrelated
13907	Myocardial infarction	18/08/2011	Unrelated
20101	probably by cerebral infarction (oral information from GP)	14/07/2011	Unrelated

Note: The adjudication regarding relatedness was carried out by an independent reviewer. Unknowns were classified as unrelated for the analysis.

Treatment allocated: Prednisolone			
**Patient number**	**Cause of death**	**Date of death**	**At least possibly related to trial treatment?**
1604	Frailty, old age and hypertension.	27/03/2012	Unrelated
4804	Site still unable to establish a cause of death - family said collapsed at home, taken to A&E and died.	14/04/2010	Unrelated
4905	1. PCP Pneumonia, 2. Bullous Pemphigoid, 3. Immunocompromised	15/06/2012	Related
5519	Chest infection and sepsis and acute kidney injury.	19/07/2013	Related
5704	Cause of death unknown - died of old age in a nursing home - all that is known	06/08/2010	Unknown
7608	Chest infection	13/03/2013	Related
8711	1. Pulomonory thrombo-embolus due to DVT, secondary to ischaemic heart disease. With contributing factor of COPD	14/03/2012	Unrelated
8907	ESBL (E-COLI) SEPSIS	21/09/2011	Related
9601	Bowel perforation - Presented with severe dehydration and seizure and next day moved to ITU with hypertension, acute renal failure and streprococcal seticaemia. Follow-up informtion - Patient 09601 admitted with dehydration and seizure, then he developed acute renal failure, streptococcus septicaemia, then bowel obstruction. Had MRI done and it was suggestive of Demyelinating encephalitis. Then he had bowel perforation and died.	29/05/2009	Related
9607	Ruptured abdominal aortic aneurysm	20/10/2009	Unrelated
9612	Sepsis cellilitis . Bp responded well to Pred. When completely withdrawn (acc to protocol) BP flared. Prednisolone 15mg od was re-introduced.Patient was on prednisolone 15mg od when cellulitis developed. Sepsis developed in spite of systemic antibiotics	05/04/2011	Related
9617	Sepsis, pneumonia. BP responded well to pred. Patient developed later pneumina and sepsis (staphylococcus aureus). Was on Vancomycin and Benzylpenicillin (past medical history of Ca Prostate, AF on warfarin, pulmonary hypertension and CVA.)	02/03/2012	Related
9619	The coroner office informed me of his cause of death which is listed as Hypothermia Hypertensive disease and pneumonia.	25/09/2012	Unrelated
10603	Pneumonia, bullous pemphigoid, chronic kidney disease, congestive cardiac failure, peripheral vascular disease, type 1 diabetes.	24/03/2010	Related
12103	Chronic renal failure (pre-existing condition)	11/11/2010	Unrelated
13901	Death certificate notes - frailty of old age	03/04/2009	Unrelated
15401	Bronchpneumonia with preceding confusion. Comorbidities included COPD and frailty	05/01/2011	Related
17402	1a. DVT, 1b. DVT, 2. diabetes mellitus, also diabetic staph.aureus skin pustules and poss. Ischaemic heart disease.	04/01/2010	Unrelated
17714	Pneumonia + COPD + pulmnary hypertension	30/04/2013	Related
17803	Ascending cholangitis	12/08/2009	Related

Note: The adjudication regarding relatedness was carried out by an independent reviewer. Unknowns were classified as unrelated for the analysis.

Figure 1: Kaplan Meier survival plot showing time to death

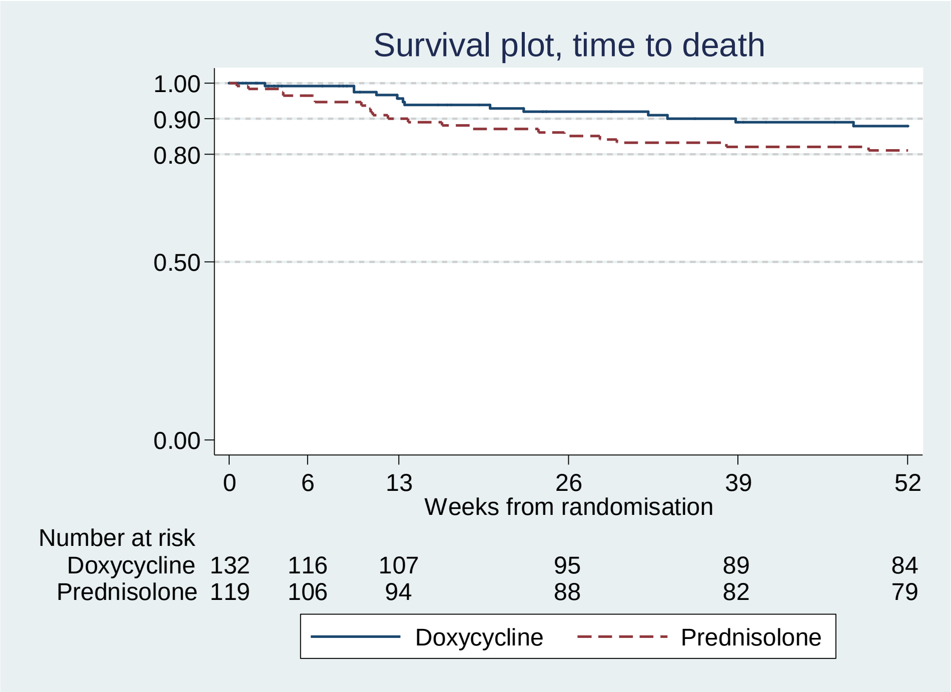

Table 10: Difference in EQ-5D scores (raw data) – mITT analysis

	Treatment					
	Doxycycline (n=110)			Prednisolone (n=101)		
	Median (IQR)	Median change from baseline	Total patients with data	Median (IQR)	Median change from baseline	Total patients with data
Baseline	0·656 (0·273 – 0·796)	0	110	0·656 (0·273 – 0·760)	0	101
Week 6	0·620 (0·353 – 0·805)	-0·036	108	0·746 (0·587 – 1·000)	+0·090	96
Week 13	0·710 (0·450 – 1·000)	+0·054	96	0·779 (0·639 – 0·925)	+0·123	92
Week 26	0·746 (0·587 – 1·000)	+0·090	85	0·796 (0·638 – 0·850)	+0·140	80
Week 39	0·727 (0·587 – 1·000)	+0·071	79	0·710 (0·587 – 1·000)	+0·054	77
Week 52	0·746 (0·587 – 1·000)	+0·090	78	0·727 (0·587 – 1·000)	+0·071	74

Table 11: Difference in DLQI scores (raw data) – mITT analysis

	Treatment					
	**Doxycycline (n=108)**			**Prednisolone (n=101)**		
	Median (IQR)	Median change from baseline	Total patients with data	Median (IQR)	Median change from baseline	Total patients with data
Baseline	10 (6-15)	0	108	11 (6-14)	0	101
Week 6	5 (2-9)	-5	106	1 (0-3)	-10	96
Week 13	2 (1-6)	-8	98	1 (0-4)	-10	90
Week 26	2 (0-4)	-8	86	1 (0-3)	-10	80
Week 39	1 (0-4)	-9	80	1 (1-3)	-10	77
Week 52	1 (0-3)	-9	79	1 (0-3)	-10	75

Figure 2: Kaplan Meier survival plot showing time to change of treatment

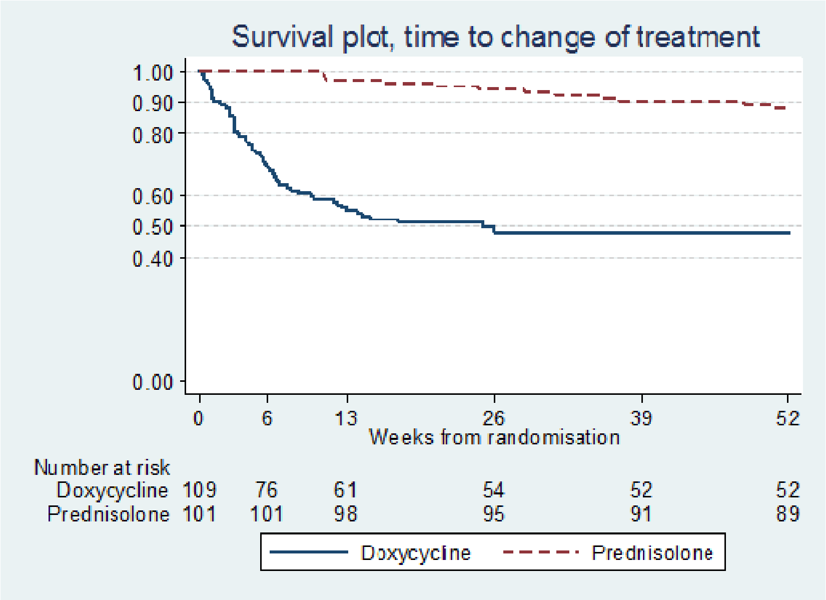

.

Note: A change in treatment refers to a change from the allocated treatment (either doxycycline or prednisolone) to the other. This graph involves the 213 patients who form the IIT population for the primary efficacy outcome

Table 12: Action taken in response to disease relapse

Action taken in response to disease relapse	Treatment allocation	
	**Doxycycline (n=37)**	**Prednisolone (n=39)**
Treatment changed to prednisolone	13 (35.1%)	0
Treatment changed to doxycycline	0	0
Dose of prednisolone increased	16 (43.2%)	30 (76.9%)
Dose of doxycycline increased	3 (8.1%)	0
Prednisolone restarted	3 (8.1%)	8 (20.5%)
Doxycycline restarted	2 (5.4%)	0
Other treatment change	0	1 (2.6%)

Note: This table refers to changes made at the point of relapse. In some instances where a patient is allocated to a particular treatment, the action may be to increase the dose or restart the unallocated drug. This is because the patient may have already changed treatment to an unallocated treatment prior to relapse, due to prior treatment failure for example.

Trial Steering Committee (TSC): Independent members: Professor Jonathan Barker (Chair), Professor Pascal Joly (Clinical expert), Dr Jonathan Leonard (Clinical expert), Ms Helena Haywood (dermatology nurse). **Patient representatives:** Penny Standen, Brian Lockwood. **Non-independent members** Professor Hywel Williams (Chief investigator), Professor Fenella Wojnarowska (Lead clinician), Dr Gudula Kirtschig (Clinical expert & co-ordinator for European sites), Professor Andrew Nunn (Senior Trial statistician), Daniel Bratton, Sunita Rehal, Thomas Godec (Trial statisticians), Dr Karen Harman (PI representative), Dr Phillip Hampton (PI representative), Dr Joanne Chalmers (research fellow) The current trial manager was also a non-independent member of the TSC.

Data Monitoring Committee: Prof Sallie Lamb (Chair), Dr Robin Graham-Brown (Independent member), Dr Tracey Young

Trial Management group: Professor Hywel Williams (Chief investigator), Professor Fenella Wojnarowska (Lead clinician), Dr Gudula Kirtschig (Clinical expert & co-ordinator for European sites), Professor Andrew Nunn (Senior Trial statistician), Daniel Bratton, Sunita Rehal, Thomas Godec (Trial statisticians), Professor James Mason (health economist). The current trial manager was also a member of the TMG.

Senior trial managers: Margaret Childs, Diane Whitham; **Trial managers:** Caroline Onions, Dr Katharine Foster, Dr Anna Sandell; **Data managers, trial co-ordinators and administrators** Daniel Simpkins, Aisha Shafayat, Robert Allen, Aimee Tooley, Sally Kucyj

List of recruiting sites

UK: Aintree University Hospitals NHS Foundation Trust, Blackpool, Fylde and Wyre Hospitals NHS Foundation Trust, Bradford Teaching Hospitals NHS Foundation Trust, Brighton and Sussex University Hospitals NHS Trust, Cambridge University Hospitals NHS Trust, City Hospitals Sunderland NHS Foundation Trust, County Durham and Darlington NHS Foundation Trust, Derby Hospitals NHS Foundation Trust, The Dudley Group of Hospitals NHS Foundation, East Kent Hospitals University NHS Foundation Trust, Frimley Park Hospital NHS Foundation Trust, Guy's and St Thomas' NHS Foundation Trust, Harrogate and District NHS Foundation Trust, Hull and East Yorkshire Hospitals NHS Trust, The Ipswich Hospital NHS Trust, James Paget University Hospitals NHS Foundation Trust, Mid Staffordshire NHS Foundation Trust, Norfolk & Norwich University Hospitals NHS Trust, North Cumbria University Hospitals NHS Trust, Northern Devon Healthcare NHS Trust, Nottingham University Hospitals NHS Trust, Oxford Radcliffe Hospitals NHS Trust, South London Healthcare NHS Trust, The Royal Berkshire NHS Foundation Trust, Royal Devon & Exeter NHS Foundation Trust, Royal Liverpool and Broadgreen University Hospitals NHS Trust, Royal United Hospitals Bath NHS Trust, Sandwell and West Birmingham Hospitals NHS Trust, Sheffield Teaching Hospitals NHS Foundation Trust, Sherwood Forest Hospitals NHS Trust, South Devon Healthcare NHS Foundation Trust, South Tees Hospitals NHS Trust, South Warwickshire General Hospitals NHS Trust, St George's Healthcare NHS Trust, Great Western Hospitals NHS Foundation Trust, Somerset Community Health, Charter House, Yeovil (incorporating Bridgewater Community Hospital), Newcastle Upon Tyne Hospitals NHS Foundation Trust, Whittington Health, United Lincolnshire Hospitals NHS Trust, University Hospitals Coventry & Warwickshire NHS Trust, University Hospitals Bristol NHS Foundation Trust, University Hospitals of Leicester NHS Trust, Whipps Cross University Hospital NHS Trust, Yeovil District Hospital NHS Foundation Trust, Grampian Health Board, Gwent Healthcare NHS Trust, Hywel Dda NHS Trust, Abertawe Bro Morgannwg University NHS Trust, Cardiff and Vale NHS Trust, Tayside Health Board.

Germany: Hautklinik der Medizinische Fakultat Carl Gustav Carus, Dresden; Department of Dermatology, University Hospital of Erlangen, Erlangen; Hautklinik des Universitätsklinikums Schleswig-Holstein, Lübeck; Hautklinik der Johannes Gutenberg-Universitat, Mainz; Hautklinik des Universitätsklinikums Münster, Münster; Hautklinik des Universitätsklinikums Schleswig-Holstein, Kiel; Hautklinik des Universitätsklinikums Würzburg, Würzburg
